# Pd single-atom-site stabilized by supported phosphomolybdic acid: design, characterizations and tandem Suzuki–Miyaura cross coupling/nitro hydrogenation reaction†

**DOI:** 10.1039/d2na00559j

**Published:** 2022-09-20

**Authors:** Jay R. Patel, Anjali U. Patel

**Affiliations:** Polyoxometalates and Catalysis Laboratory, Department of Chemistry, Faculty of Science. The Maharaja Sayajirao University of Baroda Vadodara Gujarat India anjali.patel-chem@msubaroda.ac.in

## Abstract

Herein, a single-metal (Pd) site with high surface energy was stabilized and dispersed on a support (zirconia) *via* a stabilizing agent (phosphomolybdic acid) using a wet chemistry method. HRTEM and HAADF-STEM showed a highly uniform dispersion of Pd SASc on PMA/ZrO_2_. The Pd SASc showed superior catalytic activity (>99% conversion) for the Suzuki–Miyaura cross-coupling reaction, which was further feasible for catalyzing mechanistically different nitro hydrogenation reactions in tandem fusion under mild reaction conditions. This catalyst showed outstanding activity (100% conversion and 99% selectivity) with a substrate/catalyst ratio of 927 and TON of 918 using a very low amount of Pd (0.94 × 10^−3^ mmol) for the tandem Suzuki–Miyaura cross-coupling/nitro hydrogenation reaction. It also exhibited superior stability and reusability for up to three cycles without any change in its activity.

## Abstract 2

Herein, a single-metal (Pd) site with high surface energy was stabilized and dispersed on a support (zirconia) *via* a stabilizing agent (phosphomolybdic acid) using a wet chemistry method and used as a catalyst for tandem reaction.

## Graphical abstract

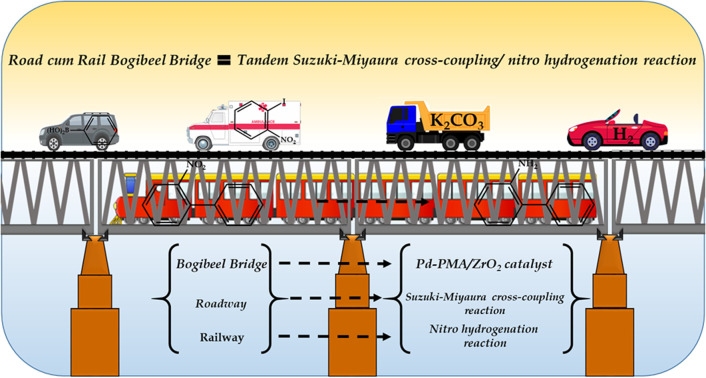

## Introduction

Single-atom site catalysts (SASc)^1–4^ refer to automatically dispersed single-metal atoms, isolated metal atoms or ions, molecular complexes, and even clusters located discretely on solid supports in the same way, which behave similarly in catalysis. SASc have attracted considerable attention in the frontier heterogeneous catalysis community because of their promising atom utilization efficiency and unique catalytic activity towards the targeted product selectivity, which play a vital role in industrial chemical synthesis.^1–6^ In addition, SASc have bridged the gap between homogeneous and heterogeneous catalysis by taking inherent advantage of both along with 100% atom utilization, high stability, easy separation from the reaction medium, and recyclability.^6–8^

However, reducing the size of metal nanoparticles down to the single-atomic level enhances their surface energy with high mobility,^1,3,9^ which leads to agglomeration by particle coalescence or by Ostwald ripening.^10,11^ Thus, to prevent this, the support or host material must have specific anchoring sites, such as coordinatively unsaturated surface atoms (*e.g.*, O^2−^ and OH^−^), surface vacancies, and heteroatom dopants (*e.g.*, N, P, S, and halogens),^12–14^ which can stabilise the catalytic metal centres as individual atoms *via* physical or chemical methods.^6,15^ However, to achieve this, physical methods such as mass-selected soft landing,^6,16^ defect engineering,^17^ iced-photochemistry,^18^ atomic layer deposition,^19^ galvanic replacement,^20^ high-temperature migration,^21^ and high-temperature pyrolysis^22,23^ are not feasible because of their low yield, and they require highly expensive and complex equipment.^6^ Alternatively, wet-chemistry techniques such as impregnation,^24^ ion-exchange,^25^ co-precipitation^26^ and adsorption methods are more practical to anchor metal atoms on the support *via* covalent interactions, ionic interactions, or geometric enclosure in small pores^12^ and avoid their aggregation during the post-treatment processes. In this direction, to date, there are several reports for the successful fabrication of SACs on various supports, such as metal hydroxide/oxides,^27–30^ graphene,^31–34^ porous nitrogen-doped carbon,^35–40^ metal–organic frameworks (MOFs),^41–45^ and zeolites.^46^

**Fig. 1:**
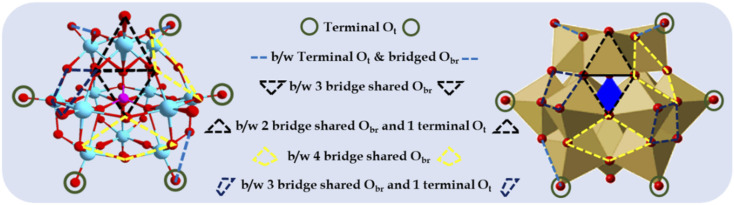
Different types of surface O atoms and possible anchoring sites for Pd atoms on Keggin-structured anion phosphomolybdic acid.

To this end, metal oxide clusters, particularly polyoxometalates (POMs) have attained considerable attraction in recent years.^1,10–12^ POMs are well-defined anionic inorganic complexes comprising molecular-level transition metal oxide clusters and synthesised by polymerizing mononuclear oxoanions (metal oxide) *via* dehydration at lower pH. Keggin-type POMs are a classic example of POMs, consisting of a highly stable 3D-cage-like structure (Fig. 1) with the anionic molecular formula of [XM_12_O_40_]^*n*−^ (X = B, Si, and P; M = Mo and W). They contain PO_4_ as the central atom surrounded by 12 hexagonal MO_6_ geometrical units with 6 nonbonding terminal oxygen atoms, resulting in technologically important fascinating physical and chemical properties. Their robust oxoanionic nature, Brønsted acidity, reversible redox behaviour, high stability, and reducing and encapsulating ability make them ideal model systems to explore and learn how to stabilize metal oxide-anchored SASc.

Based on their structural and chemical diversity, Keggin POMs are mainly divided into two classes for stabilizing SASc.^12^ Class I-POM-SACs consist of a plenary structure of [(XM_12_O_40_)^*n*−^] units, which stabilise the single atom outside the shell and possess geometrically predefined positions for an oxo-coordination environment. Class II-POM-SACs are based on a lacunary [(XM_12-m_O_40-m_)^*n*−^] unit, in which a single atom is stabilised in the lacuna created by removing one or more than one metal-oxo unit from their plenary structure. The class I-stabilized POM-SACs (Fig. 1) contain a single corner site (terminal O_t_), bridge site (b/w terminal O_t_ and bridged O_br_), three-fold hollow sites (*i.e.*, b/w three bridge-shared O_br_ and b/w two bridge-shared O_br_ and one terminal O_t_), and four-fold hollow sites (*i.e.*, b/w four bridge shared O_br_ and b/w two bridge-shared O_br_ and two terminal O_t_), making these POMs ideal stabilizers for SACs.^10,11^ However, the most preferable site is expected to be the terminal sites, as reported by Yan's group.^10,11^

In this direction, for the first time, in 2016, Yan *et al.* reported the preparation of a phosphomolybdic acid-stabilised platinum_1_ single-atom catalyst supported on activated carbon with 0.98 wt% loading of Pt and its catalytic efficiency for the hydrogenation of nitrobenzene and cyclohexanone.^10^ Based on DFT calculations, they also confirmed that the four-fold coordination site is highly favourable for stabilizing Pt SACs. Three years later, in 2019, the same group reported the synthesis of a series of highly dispersed Pt single atoms stabilized by different polyoxometalates (PTA, PMA, STA, and SMA) supported on graphene for the hydrogenation of propene.^11^ They performed DFT calculations and found that the Pt adsorption energy difference between the different POMs followed the order of Pt_1_/PTA > Pt_1_/PMA > Pt_1_/STA > Pt_1_/SMA. In 2021, Li *et al.* reported the diboration of phenylacetylene *via* a Pt SASc catalyst stabilised by an MOF fabricated using phosphomolybdic acid.^1^ However, there is no report in the literature on the synthesis and detailed characterization of phosphomolybdic acid-stabilised Pd single-atom site catalysts (SASc). It was also observed from our literature survey that few reports are available on the synthesis of functionalized aminobiphenyl *via* the tandem Suzuki–Miyaura cross coupling-nitro hydrogenation reaction over Pd-based catalysts. In 2008, for the first time, Wang *et al.* reported that Pd(OAc)_2_ catalysed the Suzuki cross-coupling reaction with the simultaneous reduction of the nitro-to amino-group in the presence of K_2_CO_3_ and DABCO as the ligand in DMF/H_2_O solvent at 150 °C and atmospheric pressure.^47^ Later, in 2011, Sullivan *et al.* synthesized a mesoporous silicabis(ethylsulfanyl)propane palladium catalyst for hydrogenation and novel one-pot two-step Suzuki cross-coupling followed by hydrogenation at room temperature for 6 h under 10 bar hydrogen pressure.^48^ In 2014, Mukherjee *et al.* reported the synthesis of a hydroquinone-based [Pd(H_2_L)(Cl)_2_] complex as an efficient room-temperature catalyst for the reduction of nitroarenes in water as the solvent and as a tandem catalyst for Suzuki–Miyaura cross coupling in ethanol followed by the reduction of nitroarenes in one pot using NaBH_4_ for the hydrogen source at room temperature.^49^ In the following year (2015), Jain *et al.* reported the dual role of mannose as a Pd stabilizing ligand during the reaction and hydrogen source for nitro reduction in the tandem cross coupling-nitro reduction reaction under microwave irradiation using a DMF/water system at 130 °C for 60 min.^50^ In the next year (2016), Pitchumani *et al.* reported the synthesis of magnetically recyclable Pd cNP/C@Fe_3_O_4_ and its catalytic activity was utilised to conduct nitro reduction, Suzuki–Miyaura coupling and sequential reactions.^51^ For the nitro reduction and sequential reactions after completion of C–C coupling, N_2_H_4_·H_2_O was used as the hydrogen source at 70 °C for 1 h. In 2018, Gabor *et al.* reported the use of the Fe_3_O_4_@Pd/PDA catalyst for Suzuki cross-couplings and tandem Suzuki cross-coupling/catalytic transfer hydrogenation sequences using formic acid as the internal hydrogen source at 80 °C for 1 h.^52^ However, it is worth noting that these catalysts have several drawbacks, such as use of a high catalyst loading, organic solvents (DMF and IPA), reducing agent (NaBH_4_ and N_2_H_4_·H_2_O), tedious isolation, and deactivation of the catalyst after regeneration.

Considering the importance of SASc and the tandem Suzuki–Miyaura cross coupling-nitro hydrogenation reaction, herein, for the first time, we report the synthesis of a zirconia-supported phosphomolybdic acid-stabilised Pd-single atom site catalyst using a simple wet chemistry method (Pd-PMA/ZrO_2_). X-ray photoelectron spectroscopy (XPS) revealed that Pd-PMA/ZrO_2_ contained Pd(0). HRTEM and dark-field scanning transmission electron microscopy (HAADF-STEM) confirmed the presence of automatically dispersed Pd atoms. Subsequently, the efficiency of this catalyst was evaluated for the Suzuki–Miyaura cross coupling and tandem Suzuki–Miyaura cross coupling-nitro hydrogenation reactions under mild reaction conditions. Furthermore, the influence of various reaction parameters such as catalyst amount, reaction time, temperature, pressure and solvent was studied thoroughly. To validate the stability of the catalyst, it was regenerated and reused for up to three cycles. The regenerated catalyst was characterised using EDX, FT-IR, and XPS and a plausible reaction mechanism for the reactions was also proposed.

## Experimental

### Materials

All chemicals used were of A.R. grade. Phosphomolybdic acid, zirconium oxychloride, ammonia, palladium chloride, 1-iodo-2-nitrobenzene, phenylboronic acid, and dichloromethane were obtained from Merck and used as received without further purification.

### Synthesis of catalyst

**Scheme 1:**
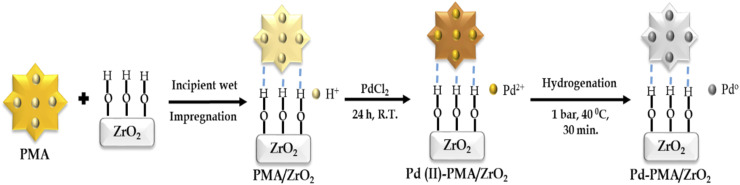
Synthesis of zirconia-supported phosphomolybdic acid-stabilized Pd SAC.

The zirconia-supported phosphomolybdic acid-stabilized Pd SAC (Pd-PMA/ZrO_2_) was synthesized *via* a wet chemistry method in three steps, as shown in Scheme 1. In step 1, hydrous zirconia (ZrO_2_) was synthesized using the previously reported method by our group.^53–55^ In step 2, PMA was supported on ZrO_2_*via* the incipient wet impregnation method, as previously reported by us.^56^ In step 3, Pd-exchanged ZrO_2_-supported phosphomolybdic acid (Pd-PMA/ZrO_2_) was synthesized by exchanging the available protons of PMA with palladium.^53–56^ For the reduction of Pd(ii) to Pd(0), the resulting wood-brown-colored material was charged in a Parr reactor under ambient conditions. The obtained grey-colored material was designated as Pd-PMA/ZrO_2_. Similarly using the wet-impregnation method, three other catalysts, *i.e.*, Pd_0.2_-PMA/ZrO_2_, Pd_0.45_-PMA/ZrO_2_ and Pd_1_-PMA/ZrO_2_, were synthesised (where 0.2, 0.45 and 1 represent the wt% of Pd). To determine the role of PMA, one more catalyst, Pd/ZrO_2_, was also synthesised using the wet impregnation method (for details, refer to the ESI†).

### Characterization

The characterization of the catalyst was carried out employing EDX, ICP, TGA, FT-IR, ^31^P MAS NMR, XPS, BET, powder XRD, TEM, HRTEM and HAADF-STEM. The details of the instruments can be found in the ESI.†

### Catalytic activity

#### Suzuki–Miyaura cross-coupling reaction

**Scheme 2:**
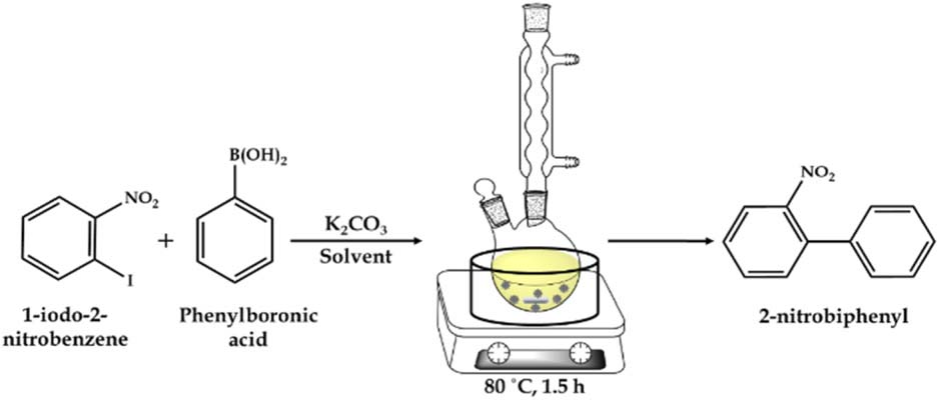
Schematic illustration of the Suzuki–Miyaura cross-coupling reaction.

The Suzuki–Miyaura (SM) cross-coupling reaction was carried out in a 50 mL glass batch reactor on a hot-plate magnetic stirrer, as shown in Scheme 2. The glass batch reactor was filled with aryl halide, phenylboronic acid, K_2_CO_3_ and EtOH : H_2_O as the solvent and catalyst. The reaction was carried out at the appropriate temperature and time with stirring in an oil bath (Scheme 2). After completion of the reaction, the reaction mass was cooled and the organic phase was extracted with dichloromethane. The organic phase was dried with anhydrous magnesium sulfate and analyzed by gas chromatography (Shimadzu-2014) using a capillary column (RTX-5). The products were confirmed by comparison with the standard samples.

#### Tandem Suzuki–Miyaura cross-coupling/nitro hydrogenation reaction

**Scheme 3:**
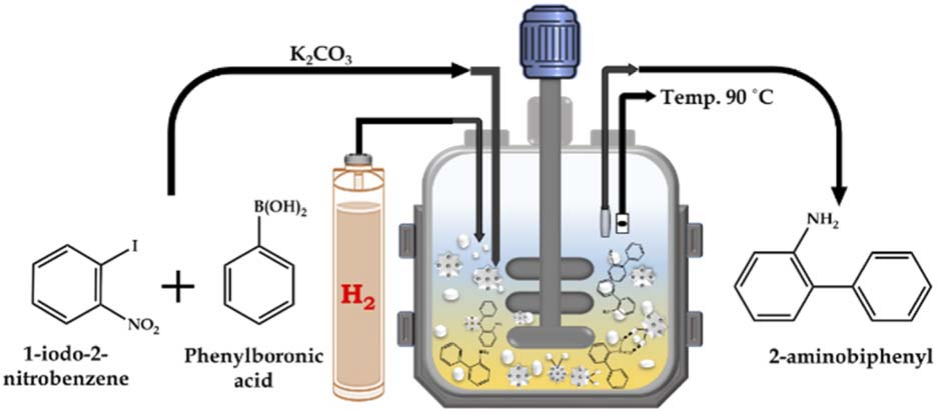
Schematic illustration of tandem Suzuki–Miyaura cross-coupling/nitro hydrogenation reaction.

The catalytic reaction was performed in a high-pressure autoclave reactor consisting of three major components, *i.e.*, 100 mL capacity batch-type reactor, H_2_ reservoir and electronic temperature and pressure controller made of SS-316. The reactor vessel was filled with aryl halide, phenylboronic acid, K_2_CO_3_, EtOH : H_2_O as the solvent and catalyst for the SM cross-coupling reaction. After completion of the SM cross-coupling, for the hydrogenation reaction, the presence of air in the unfilled space of the reactor vessel was removed by flushing with H_2_ gas several times. Finally, an appropriate amount of H_2_ pressure was applied for the reaction, at the SM cross-coupling reaction temperature with a stirring rate of 500 rpm. The continuous reduction of pressure in the vessel was used to determine the progress of the reaction. After completion, the reaction mixture was cooled to room temperature, and then the H_2_ pressure was released from the vent valve. A schematic representation of the catalytic methodology is presented in Scheme 3. The organic layer was extracted using dichloromethane, while the catalyst was collected from the liquid phase junction, and finally recovered through centrifugation. The organic phase was dried with anhydrous magnesium sulfate and analyzed by gas chromatography (Shimadzu-2014) using a capillary column (RTX-5). The products were confirmed by comparison with the standard samples.

## Results and discussion

### Characterization

In the synthesized Pd-PMA/ZrO_2_, the amount of Pd (0.43 wt%) was determined by gravimetric analysis of the standard solution and filtrate.^53–55^ The EDX elemental mapping of Pd-PMA/ZrO_2_ (Fig. S1†) showed presence of all the expected elements, *i.e.*, P, Pd, Mo, O, and Zr. The analytical values (Table 1) of Pd and Mo by EDX were found to be 0.45 wt% and 14.01 wt%, respectively, which are in good agreement with the calculated values of Pd (0.43 wt%) and Mo (14.05 wt%). The low % of Pd indicates that only the protons of PMA were exchanged. Table 1 also shows that the obtained EDX values of Pd and Mo in Pd_0.2_-PMA/ZrO_2_, Pd_0.45_-PMA/ZrO_2_ and Pd_1_-PMA/ZrO_2_ are consistent with the theoretical values. It is interesting to note that all the catalysts consist of <1% Pd, which is the first indication of the presence of a Pd single-atom site catalyst.^10,11^

**Table 1:** EDX analysis

Catalyst	EDX value (wt%)		Theoretical value (wt%)	
	Pd	Mo	Pd	Mo
Pd-PMA/ZrO_2_	0.45	14.01	0.43	14.05
Pd_0.2_-PMA/ZrO_2_	0.19	14.92	0.2	14.9
Pd_0.45_-PMA/ZrO_2_	0.44	13.9	0.45	13.9
Pd_1_-PMA/ZrO_2_	0.99	13.49	1	13.5

To support our observation, the SM cross-coupling reaction was carried out using all four synthesised catalysts and the obtained results are presented in Table 2. It can be observed from Table 2 that for all the catalysts (entries 1–4), the % conversion remained the same except for entry 1. This interesting unique observation prompted us to conduct another set of reactions (Table 2) with the same concentration of active Pd (entries 5–7) to achieve the maximum % conversion. The above study clearly indicated the presence of Pd as a single-atom site catalyst (SASc). It was also observed that the minimum amount of active species of 0.09 mg was required for the maximum conversion.

**Table 2:** Effect of Pd amount^a^

Entry	Catalyst	Catalyst amount (mg)	Active amount of Pd, mg (mmol)	% Conversion
1	Pd_0.2_-PMA/ZrO_2_	20	0.04 (0.37 × 10^−3^)	73
2	Pd-PMA/ZrO_2_	20	0.09 (0.84 × 10^−3^)	>99
3	Pd_0.45_-PMA/ZrO_2_	20	0.10 (0.94 × 10^−3^)	>99
4	Pd_1_-PMA/ZrO_2_	20	0.20 (1.87 × 10^−3^)	>99
5	*Pd_0.2_-PMA/ZrO_2_	40	0.08 (0.37 × 10^−3^)	99
6	**Pd_0.2_-PMA/ZrO_2_	45	0.08 (0.37 × 10^−3^)	>99
7	***Pd_1_-PMA/ZrO_2_	9	0.09 (0.84 × 10^−3^)	>99

a Reaction conditions: catalyst (20 mg, *40 mg, **45 mg and ***9 mg), 1-iodo-2-nitrobenzene (1.96 mmol), phenylboronic acid (2.94 mmol), K_2_CO_3_ (3.92 mmol), EtOH : H_2_O (5 : 5 mL), time (2 h), and temp. (90 °C).

It is interesting to note that the catalyst containing 0.45% Pd performed the best irrespective of the synthetic process (ion exchange by socking method or impregnation method). However, considering the reuse of the PdCl_2_ solution (filtrate), we selected the catalyst (Pd-PMA/ZrO_2_) synthesised by the ion-exchange method for further detailed characterization and catalytic study. ICP analysis of Pd-PMA/ZrO_2_ was carried out and the obtained values (0.46 wt% and 14.15 wt% for Pd and Mo, respectively), are in good agreement with that obtained theoretically and from the EDX analysis.

The thermal stability of PMA/ZrO_2_ and Pd-PMA/ZrO_2_ was determined using TGA (Fig. S2†). The TGA curve of PMA/ZrO_2_ shows (Fig. S2†) 5.3% weight loss up to 100 °C, which is attributed to the loss of adsorbed water. Further, it shows 6.4% weight loss up to 300 °C, which corresponds to the loss of crystalline water molecules present in PMA. Subsequently, the absence of further weight loss up to 500 °C indicates the stability of PMA/ZrO_2_. The TGA of Pd-PMA/ZrO_2_ showed 9.3% weight loss up to 200 °C, which should be attributed to the loss of adsorbed crystalline water molecules. Besides, no significant weight loss was observed up to 500 °C, indicating the high thermal stability of the catalyst.

**Fig. 2:**
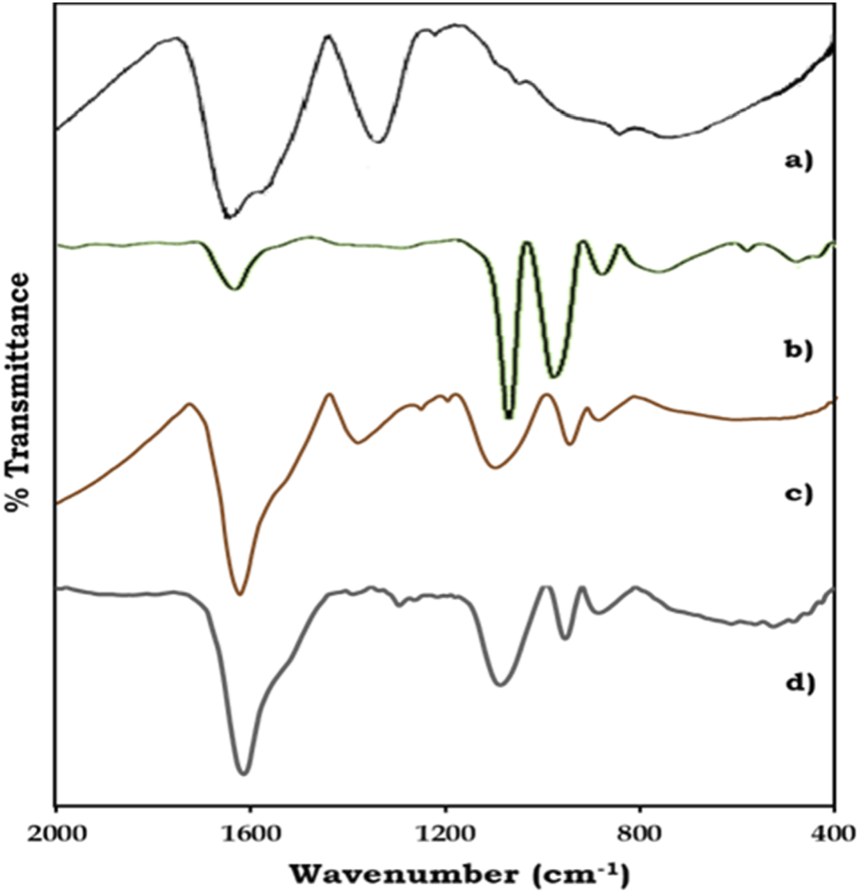
FT-IR spectra of (a) ZrO_2_, (b) PMA, (c) PMA/ZrO_2_ and (d) Pd-PMA/ZrO_2_.

The FT-IR spectra of ZrO_2_, PMA, PMA/ZrO_2_, and Pd-PMA/ZrO_2_ are displayed in Fig. 2. As shown in Fig. 2a, ZrO_2_ shows bands in the region of 1600 and 1370 cm^−1^, corresponding to H–O–H and O–H–O bending, respectively, and a broad band at 600 cm^−1^, corresponding to Zr–O–H bending.^53–56^ PMA show (Fig. 2b) four characteristic bands of the Keggin unit at 1060, 965, 870 and 790 cm^−1^, corresponding to the central P–O, terminal Mo=O, and corner-shared and edge-shared Mo–O–Mo stretching, respectively.^1,56,57^ The FT-IR spectra of PMA/ZrO_2_ (Fig. 2c) shows all the characteristic bands of PMA at 1063, 965, 870 and 790 cm^−1^ and ZrO_2_ at 1605, 1381 and 600 cm^−1^, without significant change, which indicates the retention of the Keggin unit in the synthesized material.^56^ The FT-IR spectrum of Pd-PMA/ZrO_2_ (Fig. 2d) show all the bands corresponding to PMA/ZrO_2_ (1078, 956, 878, 1605, 1381 and 600 cm^−1^) with a slight shift, which is attributed to the replacement of the counter cation protons of PMA by Pd. Here, the band corresponding to the Pd–O band (654 cm^−1^) was not observed, which may be due to its superimposition with the broad band corresponding to Zr–O–H. The obtained spectra confirm that Pd was only exchanged with the proton of PMA. If Pd was in the lacuna, splitting in the P–O band would have been observed. However, since no splitting was observed, Pd did not enter the lacuna.

**Fig. 3:**
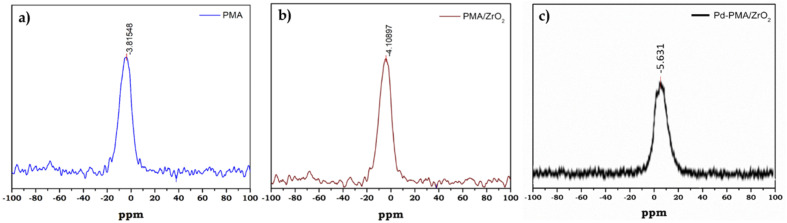
^31^P MAS NMR spectra of (a) PMA, (b) PMA/ZrO_2_ and (c) Pd-PMA/ZrO_2_.

^31^P MAS NMR of PMA, PMA/ZrO_2_ and Pd-PMA/ZrO_2_ was carried out to understand the chemical environment around the central phosphorus atom in PMA and the interaction of the anion with support. The ^31^P NMR spectrum of the pure PMA shows (Fig. 3a) a single intense peak at −3.8 ppm, which is in good agreement with the reported values.^57–59^ Moreover PMA/ZrO_2_ showed (Fig. 3b) a slight up-field shift in the peak at −3.8 to −4.1 ppm, which may be due to the strong chemical interaction between the terminal oxygen (O_t_) of PMA and surface of ZrO_2_.^56^ Furthermore, Pd-PMA/ZrO_2_ also showed (Fig. 3c) a significant up-field shift in the peak at −4.1 to −5.61 ppm. This is due to the change in the electronic environment of PMA as Pd gets exchanged with the counter proton of PMA/ZrO_2_, which is good agreement with the reported value.^10,11^

**Fig. 4:**
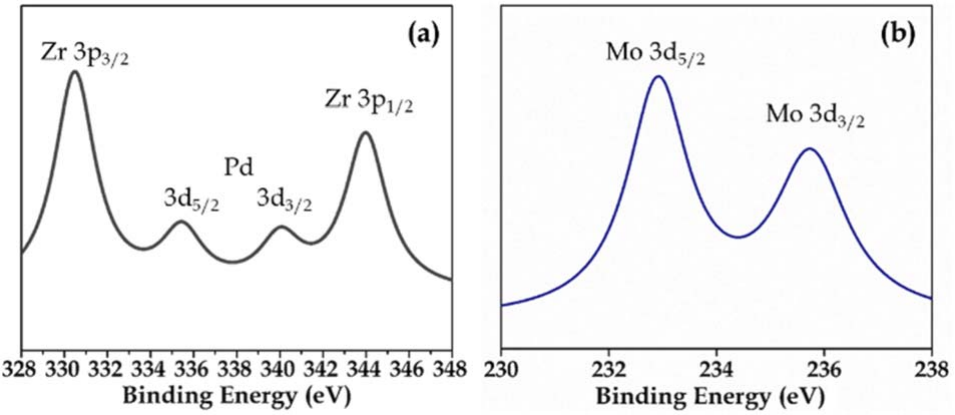
XPS of (a) Pd and (b) Mo in PMA/ZrO_2_.

XPS (Fig. S3 and S4†) of Pd-PMA/ZrO_2_ was carried out to elucidate the oxidation states of Zr, Pd, O and Mo. As shown in Fig. S3,† the two higher binding energy peaks at 331 eV and 345 eV are attributed to Zr3p_3/2_ and Zr3p_1/2_, respectively, which confirms that Zr is present in the +4 oxidation state.^53–56,60^ It also shows (Fig. S3†) a highly intense peak at 532 eV, corresponding to the O 1s spin orbit for the oxygen present in PMA and ZrO_2_. Fig. 4a displays two peaks at 334.78 eV and 340.16 eV, corresponding to two distinct spin-orbits 3d_5/2_ and 3d_3/2_, which are attributed to metallic palladium (Pd^0^),^8,53–55,61^ confirming its presence in the synthesised catalyst. Fig. 4b shows two intense peaks at 233 eV and 235 eV, corresponding to the 3d_5/2_ and 3d_3/2_ spin-orbits of Mo, respectively,^56,57^ which confirm that Mo(vi) was reduced during the reduction of Pd(ii) to Pd(0).

**Fig. 5:**
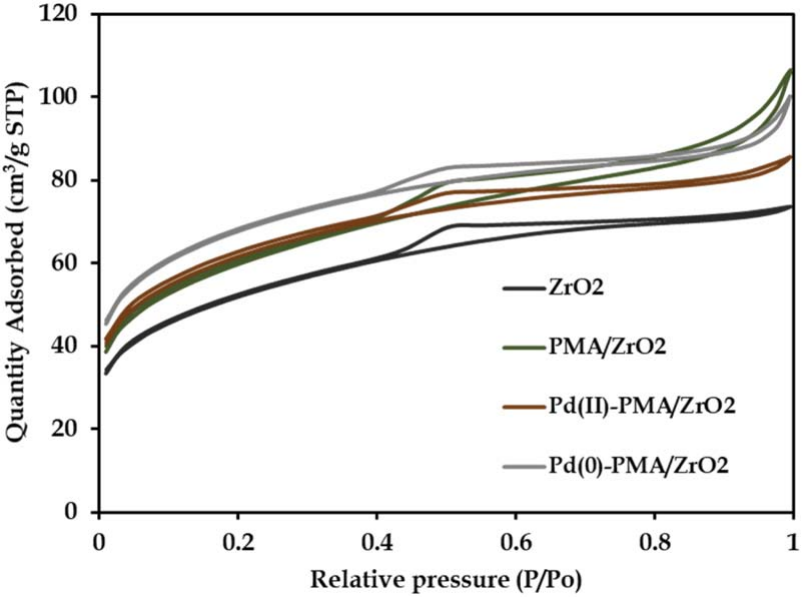
N_2_ sorption isotherms.

The N_2_ sorption isotherms of ZrO_2_, PMA/ZrO_2_, Pd-PMA/ZrO_2_ and Pd(0)-PMA/ZrO_2_ are shown in Fig. 5. The obtained unaltered nature of the N_2_ adsorption desorption isotherms for ZrO_2_ and the synthesized catalysts confirms that even after supporting PMA and the reduction of Pd on the surface of ZrO_2_, their basic structure was retained. The BET surface area of ZrO_2_ was found to be 170 m^2^ g^−1^.^53–56^ As expected, a significantly higher surface area was obtained in case of PMA/ZrO_2_ (204 m^2^ g^−1^) due to the strong interaction between PMA and ZrO_2_. However, the slight increase in surface area from 204 to 208 m^2^ g^−1^ in the case of Pd(ii)-PMA/ZrO_2_ was due to the exchange of Pd with the available protons of PMA over PMA/ZrO_2_. Further, the drastic increase in the surface area of Pd(0)-PMA/ZrO_2_ (from 208 to 228 m^2^ g^−1^)^53–55^ is because of the formation of the palladium SASc.

**Fig. 6:**
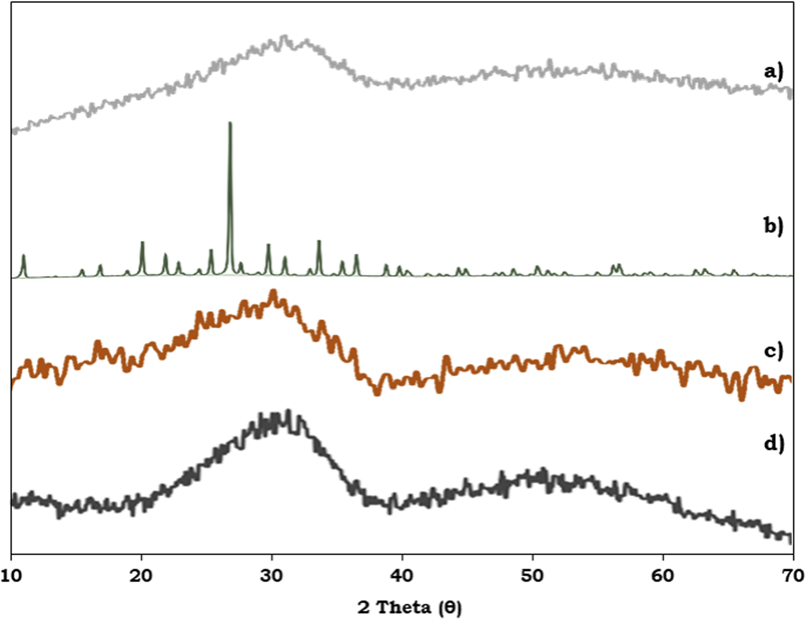
Powder XRD of (a) ZrO_2_, (b) PMA, (c) PMA/ZrO_2_ and (d) Pd-PMA/ZrO_2_.

To study the surface morphology of the ZrO_2_ support, PMA, PMA/ZrO_2_ and the synthesised Pd-PMA/ZrO_2_ catalyst, powder XRD was performed and their patterns are shown in Fig. 6. Fig. 6a shows characteristic broad peaks between 25–35°, indicating the amorphous nature of the ZrO_2_ support.^53–56^ In contrast, PMA shows (Fig. 6b) characteristic peaks in the 2*θ* range of 20° to 35°.^56,57^ The powder XRD pattern of PMA/ZrO_2_ does not show (Fig. 6c) any characteristics diffraction peaks of PMA, which indicates that PMA is highly dispersed in a non-crystalline form on ZrO_2_.^56^ The XRD pattern of Pd-PMA/ZrO_2_ (Fig. 6d) does not show any corresponding characteristic planes of Pd and is similar to that of PMA/ZrO_2_. The obtained results suggest that the amount of Pd (0.45 wt%) is beyond the detection limit of powder XRD due to its high degree of dispersion,^10,11,62^ which was further confirmed by the HRTEM and HAADF-STEM images.

**Fig. 7:**
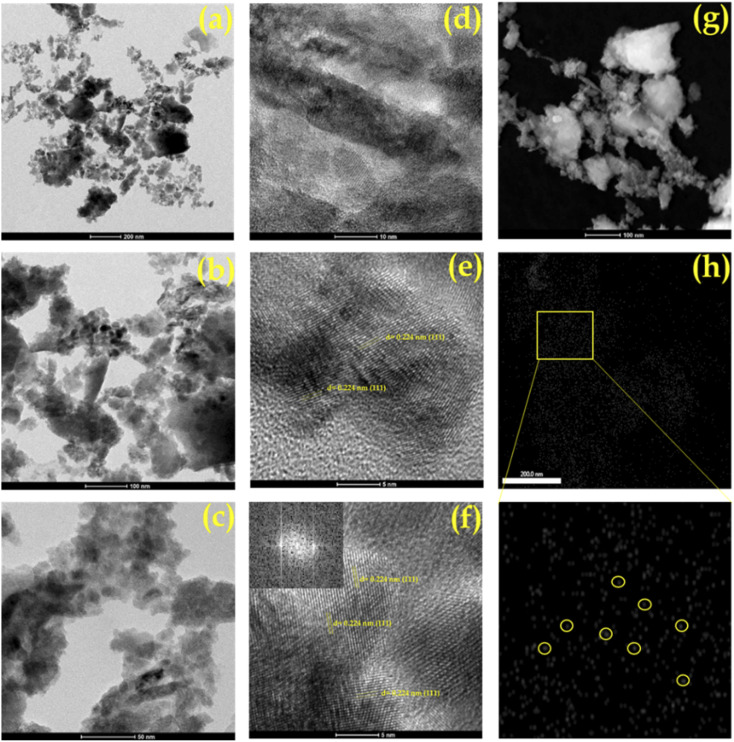
(a–c) TEM images, (d–f) HRTEM images and (g) HAADF STEM of Pd-PMA/ZrO_2_ and (h) elemental mapping of Pd.

The transmission electron microscopy (TEM) images of Pd/ZrO_2_, PMA/ZrO_2_ and Pd-PMA/ZrO_2_ at different magnifications are shown in Fig. 7. As expected, PMA/ZrO_2_ shows (Fig. S4†) a high dispersion of PMA on ZrO_2_ without agglomeration. In contrast, the TEM images of Pd/ZrO_2_ show (Fig. S5†) metallic Pd(0) nanoparticles with agglomeration. In the case of Pd-PMA/ZrO_2_, neither Pd nanoparticles nor nanoclusters can be seen in its TEM images (Fig. 7a–c). According to the EDX and ICP results, it was shown that 0.45 wt% of Pd was loaded in the synthesised catalyst, which suggests that the size of the Pd particles is beyond the detection limit of TEM.^10,11^ The high-resolution transmission electron microscopy (HR-TEM) images of Pd in Pd-PMA/ZrO_2_ (Fig. 7d–f) show a distinct lattice fringe spacing of 0.226 nm, which matches the inter planar spacing (crystallographic plane) (111) generated from the fast Fourier transform pattern of the crystal lattices produced at the diffraction spots in the horizontal direction to the alignment of the fringes.^61,63^

To verify the presence of atomically dispersed palladium single atoms, aberration-corrected high-angle annular dark-field scanning transmission electron microscopy (AC HAADF-STEM) with coupled EDX analysis measurements were performed (Fig. 7g, h and S6†). The HAADF-STEM images (Fig. 7g) show a homogeneous dispersion of Pd SACs on the surface of PMA/ZrO_2_.

Similarly, the EDX elemental mapping of Pd (Fig. 7h) clearly shows the presence of highly dispersed isolated Pd SACs without any aggregation to form nanoparticles or nano-clusters. Furthermore, the elemental overlapping images (Fig. S6†) indicate that the agglomeration of the high free surface energy Pd SACs was efficiently prevented by the electrostatic repulsive interactions derived from the PMA anions and their high surface area.

### Catalytic activity

#### Suzuki–Miyaura cross-coupling

The activity of the Pd-PMA/ZrO_2_ catalyst was evaluated for the SM cross-coupling reaction by varying different reaction parameters such as the amount of catalyst, effect of solvent, effect of base concentration, time and temperature to achieve the maximum conversion of 2-nitrobiphenyl.

**Fig. 8:**
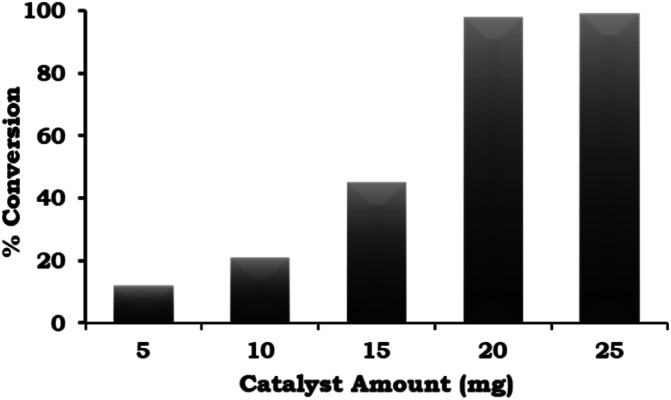
Effect of catalyst amount. Reaction conditions: 1-iodo-2-nitrobenzene (1.96 mmol), phenylboronic acid (2.94 mmol), K_2_CO_3_ (3.92 mmol), EtOH : H_2_O (5 : 5 mL), time (2 h), and temp. (90 °C).

The catalyst amount was varied from 5 mg to 25 mg and the obtained results (Fig. 8) indicate that the % conversion increased with an increase in the catalyst amount up to 20 mg because the number of Pd active site increases with respect to the substrate concentration. On further increasing the catalyst amount up to 25 mg, the % conversion remained unaltered. We assumed that the synthesised catalyst is a single atom site, and thus to confirm this, we performed an experiment to achieve maximum conversion by keeping the amount of catalyst the same (5, 10 and 15 mg) and varying the reaction time. The obtained results are shown in Table 3. It can be seen that 99% conversion was achieved on increasing the time up to 5, 4, and 3 h against each catalyst amount (5, 10 and 15 mg), which previously gave 15%, 21% and 45% conversion, respectively. Based on the obtained results, 20 mg of catalyst was employed for further optimization studies.

**Table 3:** Effect of time on % conversion^a^

Catalyst	Catalyst amount	Time	% Conversion
Pd-PMA/ZrO_2_	5	5	>99
	10	4	>99
	15	3	>99

a Reaction conditions: 1-iodo-2-nitrobenzene (1.96 mmol), phenylboronic acid (2.94 mmol), K_2_CO_3_ (3.92 mmol), EtOH : H_2_O (5 : 5 mL), and temp. (90 °C).

It is well known that the solubility of the reactant plays a crucial role in the progression of a reaction, and hence different solvents were explored while keeping the other parameters constant. As shown in Table 4, neat organic solvents such as acetonitrile, toluene, DMF and H_2_O showed negligible conversion (15%, 6%, 1% and 15%, respectively) compared to EtOH (52%). Further, to observe the on-water effect^64^ on the % conversion, the reaction was carried out in biphasic solvent systems due to their advantages, as follows: (i) aqueous water basic phase consisting ionized phenyl boronic acid and (ii) aqueous-organic phase with a lower pH-containing organic component (1-iodo-2-nitrobenzene). The SM cross-coupling reaction does not occur at the water-organic interphase, neither in water nor in the organic phase. It can be observed from the table that the reactions carried out with toluene : H_2_O and DMF : H_2_O solvent systems gave a negligible increase in the % conversion (24% and 5%, respectively). However, when the reaction was carried out with the acetonitrile : H_2_O and EtOH : H_2_O solvent systems, they gave 98% and 99% conversion, respectively. Thus, based on the obtained results, the environmentally green solvent system (EtOH : H_2_O) was selected for further study.

**Table 4:** Effect of solvent^a^

Solvent name	Solvent amount (mL)	% Conversion
Acetonitrile	10	15
Toluene	10	6
DMF	10	1
Ethanol	10	58
H_2_O	10	15
Acetonitrile : H_2_O	5 : 5	94
Toluene : H_2_O	5 : 5	24
DMF : H_2_O	5 : 5	5
EtOH : H_2_O	5 : 5	>99

a Reaction condition: 1-iodo-2-nitrobenzene (1.96 mmol), phenylboronic acid (2.94 mmol), catalyst amount (20 mg), K_2_CO_3_ (3.92 mmol), time (2 h) and temp. (90 °C).

**Fig. 9:**
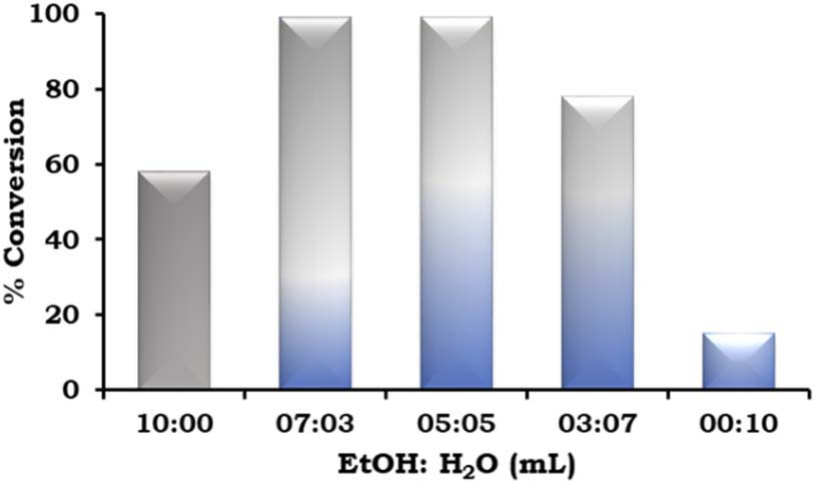
Effect of EtOH : H_2_O. Reaction condition: 1-iodo-2-nitrobenzene (1.96 mmol), phenylboronic acid (2.94 mmol), catalyst amount (20 mg), K_2_CO_3_ (3.92 mmol), time (2 h) and temp. (90 °C).

The effect of the EtOH : H_2_O ratio was also studied and the obtained results are presented in Fig. 9. According to the results, on changing the solvent system from neat ethanol to EtOH : H_2_O in different ratios (7 : 3 mL and 5 : 5 mL), there was an increase in the % conversion. This trend was observed because of the increased solubility of the base in water. However, on further changing the EtOH : H_2_O ratio (3 : 7 mL), the % conversion decreased and very low conversion was observed in neat H_2_O because of the poor solubility of the organic substrates.

**Fig. 10:**
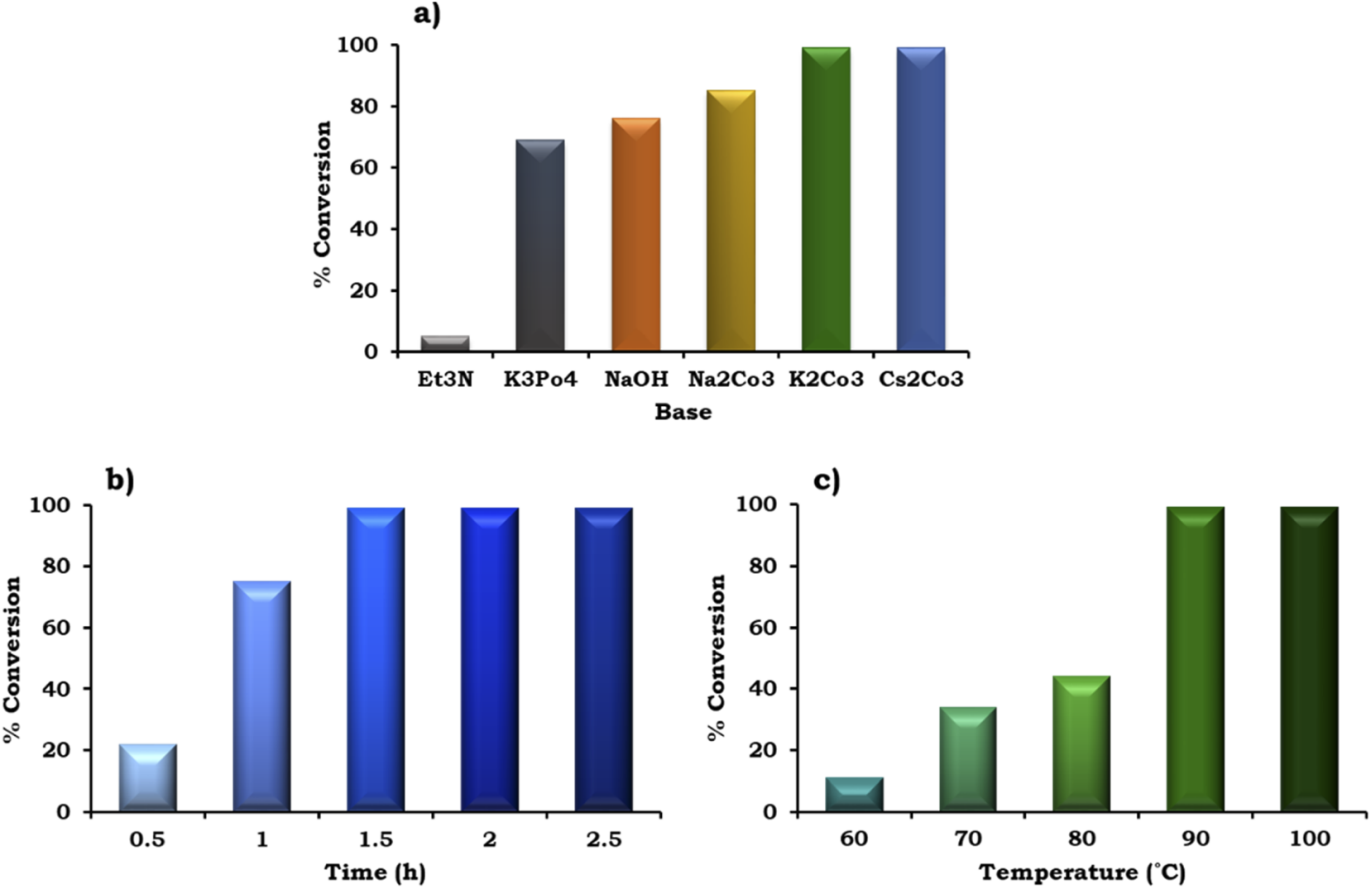
Optimization of the reaction parameters (a) effect of base. Catalyst (20 mg, active amount of Pd: 0.09 mg), 1-iodo-2-nitrobenzene (1.96 mmol), phenylboronic acid (2.94 mmol), EtOH : H_2_O (5 : 5 mL), time (2 h), temp. (90 °C). (b) Effect of time. Catalyst (20 mg, active amount of Pd: 0.09 mg), 1-iodo-2-nitrobenzene (1.96 mmol), phenylboronic acid (2.94 mmol), EtOH : H_2_O (5 : 5 mL), K_2_CO_3_ (3.92 mmol), temp. (90 °C). (c) Effect of temperature. Catalyst (20 mg, active amount of Pd: 0.09 mg), 1-iodo-2-nitrobenzene (1.96 mmol), phenylboronic acid (2.94 mmol), EtOH : H_2_O (5 : 5 mL), K_2_CO_3_ (3.92 mmol), and time (1.5 h).

The effect of various organic–inorganic bases on the reaction rate was also studied and the obtained results are shown in Fig. 10a. This study indicated that the organic base triethyl amine (Et_3_N) gave negligible conversion (5%) compared to the inorganic bases. With the use of different inorganic bases, the % conversation was found to follow the order of K_3_PO_4_ (69%) < NaOH (76%) < Na_2_CO_3_ (85%) < K_2_CO_3_ ≈ Cs_2_CO_3_ (99%). The highest conversion (99%) was obtained with K_2_CO_3_ and Cs_2_CO_3_, but for further studies we have selected K_2_CO_3_ because it is environmentally benign compared to Cs_2_CO_3_.

To achieve the highest conversion, the influence of the reaction time was also monitored and the obtained results are shown in Fig. 10b. It can be seen that with an increase in time from 0.5 h to 1.5 h, at an interval of 30 min, the % conversion increased gradually, which is in good agreement with the well-known fact that with an increase in time, there is an increase in the formation of reactive intermediates from the reactants, which results in the formation of the product. Further, on prolonging the time up to 2 h, no significant change was observed in the % conversion. Therefore, 1.5 h was considered optimal with 98% conversion for the further optimization of temperature.

The effect of temperature on the formation of the C–C bond between 1-iodo-2-nitrobenzene and phenylboronic acid was also investigated for the best catalytic performance at an interval of 10 °C in the range of 90–60 °C. As shown in Fig. 10c, on increasing the temperature, % conversion increased gradually up to 90 °C. Based on this, 90 °C temperature was considered optimal to achieve 99% conversion.

Based on this study, the optimized conditions for 99% conversion with a TON of 2294 and TOF of 1529 are as follows: 1-iodo-2-nitrobenzene (1.96 mmol), phenylboronic acid (2.94 mmol), catalyst (20 mg, active amount of Pd: 0.09 mg), EtOH : H_2_O (5 : 5 mL), K_2_CO_3_ (3.92 mmol), 1.5 h, 90 °C with substrate ratio/catalyst of 2317/1.

#### Tandem Suzuki–Miyaura cross-coupling/nitro hydrogenation reaction

Based on the excellent activity of Pd-PMA/ZrO_2_ for the SM cross-coupling of 1-iodo-2-nitrobenzene to 2-nitrobiphenyl and considering the importance of the nitro hydrogenation reaction, we thought of the idea to merge two mechanistically different catalytic transformations into a single tandem process. Initially, we applied the SM cross-coupling for 1.5 h in a high-pressure autoclave at 90 °C, and subsequently H_2_ pressure was applied and the reaction was continued for 24 h (Scheme 3).

**Fig. 11:**
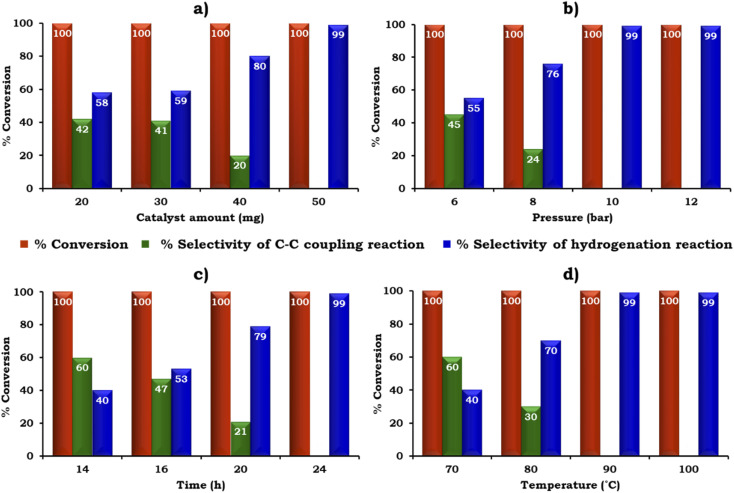
Optimization of reaction parameters: (a) effect of catalyst amount. 1-Iodo-2-nitrobenzene (1.96 mmol), phenylboronic acid (2.94 mmol), K_2_CO_3_ (3.92 mmol), EtOH : H_2_O (5 : 5 mL), pressure (10 bar), time (24 h) and temp. (90 °C). (b) Effect of pressure. Catalyst (50 mg, active amount of Pd: 0.225 mg), 1-iodo-2-nitrobenzene (1.96 mmol), phenylboronic acid (2.94 mmol), K_2_CO_3_ (3.92 mmol), EtOH : H_2_O (5 : 5 mL), time (24 h) and temp. (90 °C). (c) Effect of time. Catalyst (50 mg, active amount of Pd: 0.225 mg), 1-iodo-2-nitrobenzene (1.96 mmol), phenylboronic acid (2.94 mmol), K_2_CO_3_ (3.92 mmol), EtOH : H_2_O (5 : 5 mL), pressure (10 bar) and temp. (90 °C). (d) Effect of temperature. Catalyst (50 mg, active amount of Pd: 0.225 mg), 1-iodo-2-nitrobenzene (1.96 mmol), phenylboronic acid (2.94 mmol), K_2_CO_3_ (3.92 mmol), EtOH : H_2_O (5 : 5 mL), pressure (10 bar) and time (24 h).

The catalyst amount was screened from 20 to 50 mg to achieve the maximum conversion and selectivity for the hydrogenated product, 2-aminobiphenyl, as shown in Fig. 11a. The obtained results showed that 100% conversion was achieved with 20 mg but the selectivity for 2-aminobiphenyl was only 58%. The low selectivity for 2-aminobiphenyl obtained may be due to the difficulty in approaching the NO_2_ group of 2-nitrobiphenyl by Pd or due to the conformation of two phenyl rings. Further, on increasing the amount of catalyst to 50 mg, 100% conversion was obtained and the selectivity for 2-aminobiphenyl gradually increased because a higher amount of Pd was available to react with the reactant species. Therefore, 50 mg of catalyst was considered optimal for further studies.

The influence of H_2_ pressure plays an important role in the catalytic transfer hydrogenation reaction. Therefore, the effect of H_2_ pressure was also studied and the obtained results are shown in Fig. 11b. It can be seen that the selectivity for the hydrogenated product increased gradually on increasing the pressure from 6 to 10 bar. Based on the results, 10 bar pressure was optimal to achieve 100% conversion with 99% selectivity for 2-aminobiphenyl.

Similarly, the effect of time and temperature on the reaction rate was also investigated to achieve the maximum conversion with selectivity for 2-aminobiphenyl and the obtained data is shown in Fig. 11c and d. Based on this study, the optimized conditions for 100% conversion with 99% selectivity for 2-aminobiphenyl are as follows: iodobenzene (1.96 mmol), phenylboronic acid (2.94 mmol), catalyst (50 mg, active amount of Pd: 0.225 mg), K_2_CO_3_ (2.96 mmol), EtOH : H_2_O (5 : 5 mL), pressure (10 bar), time (24 h) and temp (90 °C) with TON (918), TOF (38) and substrate ratio/catalyst (927).

### Control experiment

To understand the role of each component in the synthesised material, control experiments were carried out with ZrO_2_, PMA, PMA/ZrO_2_, PdCl_2_, Pd/ZrO_2_ and Pd-PMA/ZrO_2_ under the optimized conditions for the SM cross-coupling and tandem Suzuki–Miyaura cross-coupling/nitro hydrogenation reactions. As shown in Table 5, ZrO_2_, PMA and PMA/ZrO_2_ are inactive towards both reactions, further confirming that PMA acts as a stabilizing agent for Pd, as we already mentioned in the introduction. However, we obtained almost same the % conversion and selectivity for both reactions when they were carried out with PdCl_2_ and Pd/ZrO_2_ having same active amount of Pd. Thus, the obtained results confirm that Pd is the only active reactive species for the progress of the reaction.

**Table 5:** Control experiment

Catalyst	% Conversion^a^	% Conversion^b^	% Selectivity	
			C–C coupling	NO_2_ hydrogenation
ZrO_2_	NA	NA	NA	NA
PMA	NA	NA	NA	NA
PMA/ZrO_2_	NA	NA	NA	NA
PdCl_2_	92	100	4	96
Pd/ZrO_2_	96	100	7	93
Pd-PMA/ZrO_2_	>99	100	NA	99

a For SM cross-coupling reaction: 1-iodo-2-nitrobenzene (1.96 mmol), phenylboronic acid (2.94 mmol), catalyst (ZrO_2_; 15.38 mg, PMA; 4.62 mg, PMA/ZO_2_; 19.1 mg), EtOH : H_2_O (5 : 5 mL), K_2_CO_3_ (3.96 mmol), time (1.5 h) and temp. (90 °C).

b For Tandem reaction: 1-iodo-2-nitrobenzene (1.96 mmol), phenylboronic acid (2.94 mmol), catalyst (ZrO_2_; 38.45 mg, PMA; 11.5 mg, PMA/ZO_2_; 47.75 mg), EtOH : H_2_O (5 : 5 mL), K_2_CO_3_ (3.96 mmol), pressure (10 bar), time (24 h) and temp. (90 °C).

### Hot filtration and heterogeneity test

In heterogeneous catalysis, one of the basic requirements is that the catalyst should retain its heterogeneity for several cycles with its active sites remaining intact. Thus, to check the leaching of Pd from Pd-PMA/ZrO_2_ and Pd/ZrO_2_ in both reactions, a hot filtration test was carried out. In the case of the SM cross-coupling reaction, initially, the reaction was performed for up to 45 min with Pd-PMA/ZrO_2_ and Pd/ZrO_2_, and afterward both catalysts were removed from the hot reaction mass and the reaction was left to further proceed for 45 min (total 90 min). Similarly, the tandem Suzuki–Miyaura cross-coupling/nitro hydrogenation reaction was carried out using both catalysts for up to 16 h. Subsequently, both catalysts were removed and the reaction was continued for up to 24 h (another 8 h). After completion of the reactions, the organic layer was extracted using dichloromethane and analysed by gas chromatography.

In the case of Pd/ZrO_2_, the obtained results (Table 6) show an increase in conversion by 8% in the SM cross-coupling reaction after removal of the catalyst. In contrast, in the one-pot reaction, no change in % conversion was observed, but a 6% increase in selectivity for NO_2_ hydrogenation after removal of catalyst was observed. The obtained results from both the reactions indicate that Pd leached from the support during the reaction. However, Pd-PMA/ZrO_2_ showed no change in % conversion after the removal of the catalyst in both reactions, which confirms that the Pd single atom does not leach during the reaction. Further the heterogeneity of Pd-PMA/ZrO_2_ was confirmed by EDX analysis of the regenerated catalyst. The retention of the Pd content (0.45 wt%) indicated that the Pd SACs was stabilized by PMA over the surface of ZrO_2_. Thus, the obtained results confirm the true heterogeneous nature of the catalyst, which was also reflected in the recycling study.

**Table 6:** Heterogeneity test of Pd/ZrO_2_ and Pd-PMA/ZrO_2_

Catalyst	% Conversion^a^	% Conversion^b^	% Selectivity	
			C–C coupling	NO_2_ hydrogenation
Pd/ZrO_2_	43 (after 1 h)	100 (after 16 h)	41	59
	51 (after 2 h)	100 (after 24 h)	36	64
Pd-PMA/ZrO_2_	51 (after 1 h)	100 (after 16 h)	46	56
	52 (after 2 h)	100 (after 24 h)	46	56

a For the SM cross-coupling reaction: 1-iodo-2-nitrobenzene (1.96 mmol), phenylboronic acid (2.94 mmol), catalyst (20 mg), EtOH : H_2_O (5 : 5 mL), K_2_CO_3_ (3.96 mmol), time (1.5 h) and temp. (90 °C).

b For tandem reaction: 1-iodo-2-nitrobenzene (1.96 mmol), phenylboronic acid (2.94 mmol), catalyst (50 mg), K_2_CO_3_ (3.96 mmol), EtOH : H_2_O (5 : 5 mL), pressure (10 bar), time (24 h) and temp. (90 °C).

### Catalyst reusability

For any chemical process, the sustainability of the catalyst up to several cycles is one of the key issues. Thus, after completion of the reaction, Pd-PMA/ZrO_2_ was regenerated for both reactions through centrifugation and washed with dichloromethane followed by water, and finally air dried for its reuse for the next catalytic run. As shown in Table 7, consistent catalytic activity occurred for up to three cycles for both reactions and the catalyst could be reused for further cycles, which depends on the choice of the chemist. This clearly shows that the synthesised catalyst is truly heterogeneous in nature.

**Table 7:** Catalyst reusability

Catalyst	% Conversion^a^	% Conversion^b^	% Selectivity	
			C–C coupling	NO_2_ hydrogenation
Fresh	>99	100	NA	99
R-1	99	100	NA	99
R-2	99	100	NA	99
R-3	99	100	2	98

a For SM cross-coupling reaction: 1-iodo-2-nitrobenzene (1.96 mmol), phenylboronic acid (2.94 mmol), catalyst (20 mg), K_2_CO_3_ (3.96 mmol), EtOH : H_2_O (5 : 5 mL), time (1.5 h) and temp. (90 °C).

b For tandem reaction: 1-iodo-2-nitrobenzene (1.96 mmol), phenylboronic acid (2.94 mmol), catalyst (50 mg), K_2_CO_3_ (3.96 mmol), EtOH : H_2_O (5 : 5 mL), pressure (10 bar), time (24 h) and temp. (90 °C).

### Characterization of regenerated catalyst

To check sustainability of the catalyst, the regenerated catalyst (R-Pd-PMA/ZrO_2_) was characterised by EDX, FT-IR, XPS and powder XRD.

**Fig. 12:**
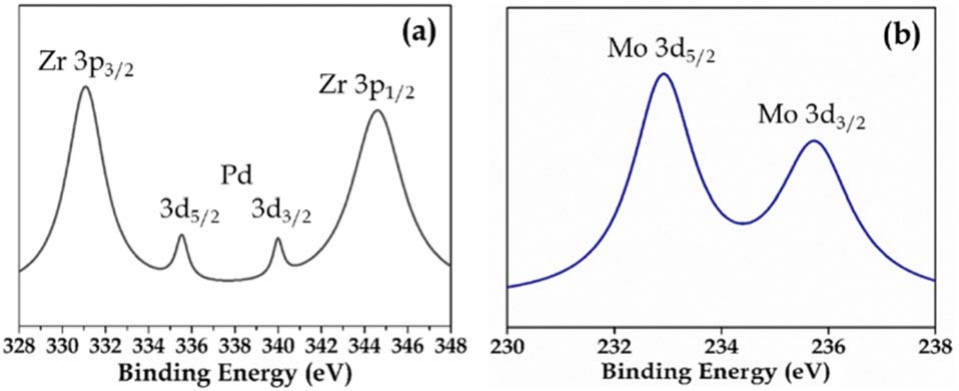
XPS of (a) Pd and (b) Mo in R-PMA/ZrO_2_.

Elemental mapping (Fig. S7†) confirmed the presence of all the elements. The EDX values of Pd (0.44 wt%) and Mo (15.08 wt%) for regenerated Pd-PMA/ZrO_2_ are in good agreement with that of the fresh catalyst (0.72 wt% Pd and 15.19 wt% Mo), which confirms that there was no leaching of the Pd atoms from the catalyst during the reaction. The FT-IR spectra of the regenerated and fresh catalysts are displayed in Fig. S8,† which are almost identical without any significant shift in the bands. This indicates the retention of the catalyst structure even after regeneration of the catalyst. However, the regenerated catalyst showed bands with a slightly lower intensity compared to that of the fresh catalyst, which may be due to the sticking of the substrates on the catalyst, but it had no effect on its efficiency. Fig. 12 shows the XPS of the regenerated catalyst (R-Pd-PMA/ZrO_2_). It consist of all the characteristic peaks of Pd, Mo, Zr and O, which are identical to that obtained for the fresh catalyst (Fig. 4). This further confirms no reduction of Mo(vi) occurred and the retention of the active Pd SACs on the catalyst surface, thus confirming the sustainability of this catalyst. The recycled catalyst (R-Pd-PMA/ZrO_2_) showed a similar powder XRD pattern to that of the fresh catalyst (Fig. S9†). The obtained results show the retention of the highly dispersed nature of the catalyst, which further confirms the sustainability of this catalyst.

### Mechanism

**Scheme 4:**
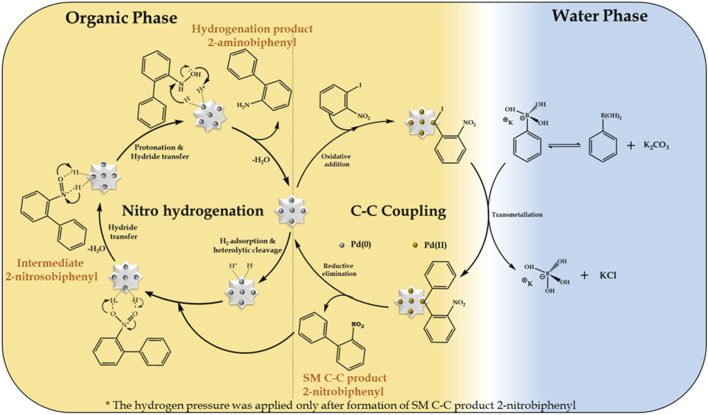
Plausible mechanism for the Suzuki–Miyaura cross-coupling/nitro hydrogenation reaction.

In the present work, we propose that the well-reported mechanism (Scheme 4) occurs for two mechanistically different catalytic transformation reactions, as follows: (i) SM C–C^54,65^ and (ii) nitro hydrogenation.^66^ The SM C–C reaction involves oxidative addition, transmetallation and reductive elimination, while in the case of the nitro hydrogenation reaction, we propose the universal hydride transfer *via* the heterolytic cleavage of H_2_.

In the case of the reaction, as mentioned for the “on-water” effect on SM C–C, organopalladium(ii) species is formed in the organic phase *via* the oxidative addition of the Pd SAC atom with 1-iodo-2-nitrobenzene. Alternatively, in the water phase, a boronate complex is formed by the ionization of phenyl boronic acid in a higher basic water phase (formed by K_2_CO_3_). Afterwards, at the water-organic interphase, the organopalladium species undergo transmetallation with the boronate complex to produce a new organopalladium complex by replacing the phenyl ring of phenylboronic acid with the halides of the organopalladium species. Finally, the product (2-nitrobiphenyl) is formed and Pd(O) SACs are regenerated for further hydrogenation reaction. For the hydrogenation reaction after applying H_2_ pressure, initially, hydrogen gets adsorbed on the Pd atom to form Pd–H by heterolytic cleavage of H_2_, followed by the adsorption of 2-nitrobiphenyl on the Pd–H surface of the catalyst by electrostatic interactions to form an anionic *N*,*N*-dihydroxylamine *via* hydride transfer. This is followed by protonation to yield an *N*,*N*-dihydroxylamine intermediate by eliminating a water molecule, which gets dehydrated to generate 2-nitrosobiphenyl. The nitroso group in 2-nitrosobiphenyl is further attacked by the hydride of Pd–H and protonated to yield *N*-hydroxylamine, which is further protonated, followed by hydride transfer to form 2-aminobiphenyl and the catalyst is regenerated for the next catalytic cycle.

Further, for the nitro hydrogenation reaction step, to confirm the formation of nitroso intermediates, a set of reactions was performed after the completion of the SM C–C reaction, where H_2_ pressure was applied for 2, 6 and 10 h and the obtained reaction mass was analysed by GC. The results showed 100% conversion with only 2%, 11% and 21% selectivity for 2-aminobiphenyl in 2, 6 and 10 h, respectively, and no other peak corresponding to 2-nitrosobiphenyl was found. This indicates that the formed 2-nitrosobiphenyl intermediate is unstable and difficult to isolate. To further confirm this, reaction mass obtained after 10 h was analysed by ^1^H NMR and the spectra show peaks corresponding to 2-nitrobiphenyl (Fig. S10†) and 2-aminobiphenyl (Fig. S11†) but no peak corresponding to 2-nitrosobiphenyl (Fig. S12†). The absence of a peak corresponding to nitroso intermediate shows that the intermediate is unstable and is immediately converted to an amine. If not, some additional peaks should be observed in the NMR spectra. Thus, this study clearly indicates that the intermediate is highly unstable, and hence difficult to isolate from the reaction mixture.

## Conclusions

In the summary, the results of this work emphasized the successful development of a novel simple strategy for the fabrication of Pd SASc stabilized by phosphomolybdic acid supported on zirconia (Pd-PMA/ZrO_2_) by an ion-exchange method. A thorough systematic characterization was carried out *via* FT-IR spectroscopy, ^31^P MAS NMR, BET, and powder XRD, which confirmed that Pd exchanged only with the available protons of PMA/ZrO_2_ with the retention of its basic structure. The metallic state of Pd was confirmed by XPS and the presence of Pd SASc was revealed by HRTEM and HAADF-STEM analysis. The synthesised catalyst exhibited outstanding catalytic activity (100% conversion and 99% selectivity for 2-aminobyhenyl) in the tandem Suzuki–Miyaura cross-coupling/nitro hydrogenation reaction under mild reaction conditions using an ethanol–water biphasic system. The control experiment and hot filtration test confirmed the role of PMA in stabilising the Pd SASc, without aggregation. We also believe that the designed strategy can be extended for the design of other precious metal-based single-atom site catalysts for various organic transformations.

## Author contributions

Jay Patel: methodology, validation, formal analysis, investigation, writing – original draft. Anjali Patel: conceptualization, validation, writing – review & editing, visualization, supervision.

## Conflicts of interest

There are no conflicts to declare.

## Supplementary Material

NA-004-D2NA00559J-s001
