# Supplementary material for: Pd single-atom-site stabilized by supported phosphomolybdic acid: design, characterizations and tandem Suzuki–Miyaura cross coupling/nitro hydrogenation reaction
Source: Nanoscale Adv. 2022 Sep 20;4(20):4321–34. doi: 10.1039/d2na00559j (PMC9552875; doi:10.1039/d2na00559j)
Supplement: NA-004-D2NA00559J-s001 [file NA-004-D2NA00559J-s001.pdf]

Electronic Supplementary Material (ESI) for Nanoscale Advances.
This journal is © The Royal Society of Chemistry 2022

Supplementary Information

Pd Single-atom-Site stabilized by supported phosphomolybdic acid: design, characterizations and Tandem Suzuki–Miyaura cross coupling/ nitro hydrogenation reaction

Jay Patel and Anjali Patel*

*Polyoxometalates and Catalysis Laboratory, Department of Chemistry, Faculty of Science, The Maharaja Sayajirao University of Baroda, Vadodara-390002, India.

*E-mail: anjali.patel-chem@msubaroda.ac.in

Catalyst Synthesis

The catalyst synthesis was carried out by wet chemistry method in three steps.

Step-1: Synthesis of Zirconia (ZrO_2):
ZrO_2 was synthesized via previously reported method by our group. Briefly, 25% aqueous ammonia solution was added to 100 mL aqueous solution of $ZrOCl_2 \cdot 8H_2O$ (10 % w/v) to obtain a pH of 8.5. The resulting precipitate was aged in water bath at 100°C for 1 h, filtered and washed with conductivity water until all the free chloride was removed. The obtained solid was dried at 100 °C for 10 h and designated as ZrO_2

Step-2: Synthesis of supported phosphomolybdic acid (PMA/ZrO_2):
30% PMA/ZrO_2 synthesized by incipient wet impregnation method (Scheme S1) as reported by us earlier. 1 g ZrO_2 was impregnated with an aqueous solution of PMA (0.3/30 g mL-1 of double distilled water) and dried for 10 hours at 100 °C after evaporation of the water from a suspension. The obtained light yellowish green coloured material was designated as PMA/ZrO_2.

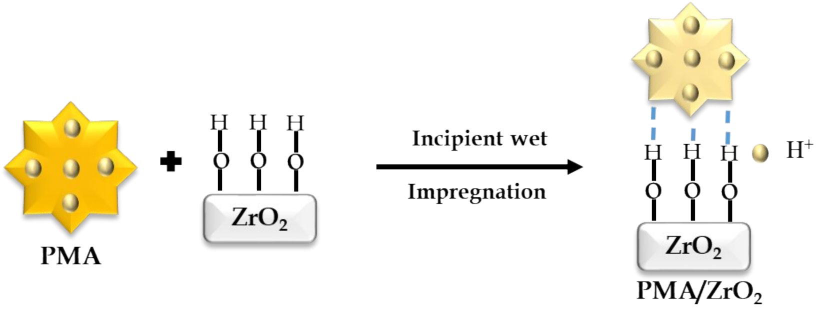

Scheme S1. Synthesis of PMA/ZrO_2

Step-3: Synthesis of Zirconia supported Phosphomolybdic acid stabilized Pd SASc (Pd-PMA/ZrO_2) by soaking and post reducing method:

Zirconia supported Phosphomolybdic acid stabilized Pd SASc was synthesized by exchanging (Scheme S2) the available protons of PMA with Palladium. 1 g PMA/ZrO_2 was soaked with 25 mL 0.05 M $PdCl_2$ aqueous solution with interment stirring for 24 hours. The mixture was filtered, washed with distilled water to remove excess Pd and dried in air at room temperature. The obtained wooden brown colored material was designated as Pd(II)-PMA/ZrO_2 . Finally, the synthesized material was charged in a Parr reactor for reduction of Pd(II) to Pd(0), under 1 bar H_2 pressure, at 40 °C for 30 min. The obtained grey colored material designated as Pd-PMA/ZrO_2.

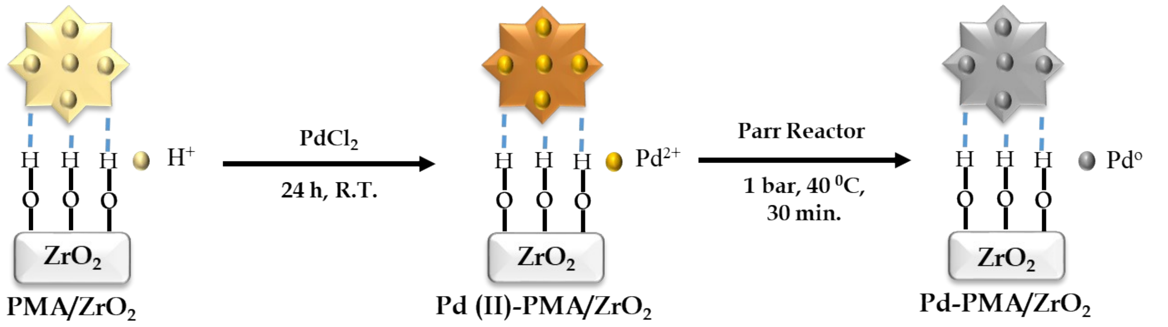

Scheme S2. Synthesis of Pd-PMA/ZrO_2

Synthesis of Pd-PMA/ZrO_2 by incipient wet impregnation method:

A series of catalyst containing of 0.2, 0.45 and 1 Wt% of Pd in Zirconia supported Phosphomolybdic acid stabilized Pd SASc were synthesised by wet impregnation method. 1 g of PMA/ZrO_2 was added to aqueous solution of $PdCl_2$ (0.00334/30, 0.00751/30 and 0.0167/30 g mL^{-1} in double distilled water) and dried for 10 hours at 100 °C. The obtained light wooden brown colour material was designated as 0.2%, 0.45% and 0.1% Pd(II)-PMA/ZrO_2 respectively. For the reduction of Pd(II) to Pd(0), the resulting martial charged in a Parr reactor under 1 bar H_2 pressure, at 40 °C for 30 min. the obtained black colored material designated as $Pd_{0.2}$-PMA/ZrO_2, $Pd_{0.45}$-PMA/ZrO2 and Pd_1-PMA/ZrO_2 respectively.

Synthesis of Pd/ZrO_2 by incipient wet impregnation method:

1 g ZrO_2 was impregnated (Scheme S3) with an aqueous solution of $PdCl_2$ (0.0085/30 g mL-1 of double distilled water) and dried for 10 hours at 100 °C after evaporation of the water from a suspension. The obtained light wooden brown colour material was designated as 1% Pd(II)/ZrO_2. To the reduce Pd(II) to Pd(0), the resulting material charged in a Parr reactor under 1 bar H_2 pressure, at 40 °C for 30 min. the obtained grey colored material designated as Pd/ZrO_2.

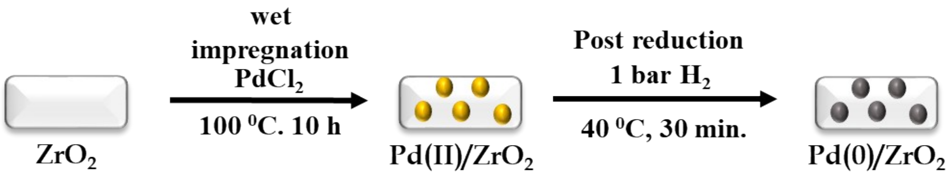

Scheme S3. Synthesis of Pd/ZrO_2

Physicochemical Characterizations

The amount of Pd was determined by gravimetric method. Energy dispersive X-ray spectra and hyperspectral element maps were acquired by JSM 5610 LV combined with INCA instrument using carbon strip. ICP of synthesized catalyst performed on Avio$^{@}$500 ICP-OES. Thermo gravimetric analysis (TGA) was performed using Mettler Toledo Star SW 7.01 up to 500 °C under nitrogen atmosphere. The samples were degassed at 75 °C for 7 h prior to analysis. FT-IR spectrum of the materials were performed by using the KBr wafer on Shimadzu instrument (IRAffinity-1S). ^{31}P MAS NMR spectra were recorded by BRUKER Avance DSX-300, at 121.49 MHz using a 7 mm rotor probe with 85% phosphoric acid as an external standard. X-ray photoelectron spectroscopy (XPS) measurements were performed with Auger Electron Spectroscopy (AES) Module PHI 5000 Versa Prob II. Nitrogen physisorption isotherms were measured through Micromeritics ASAP 2010 Surface area analyzer at -196 °C. Specific surface area was calculated using Brunauer-Emmett- Teller (BET method). Powder X-ray Diffraction (Powder XRD) was carried out using Philips Diffractometer (Model PW-1830). Transmission electron microscopy (TEM) micrographs were recorded on TEM CM 200 (Make: PHILIPS, Model: CM 200, Specification: Operating voltages: 20-200kv and Resolution: 2.4 Å). High-resolution transmission electron microscopy (HRTEM) analysis were carried out on Field Emission Gun-Transmission Electron Microscope (Resolution: Point: 0.19 nm, Line: 0.1 nm, Magnification: 50 x - 1.5 M x accelerating voltage of 200 kV; Make: JEOL; Model: JEM 2100F) with attachment systems EDS and STEM. The samples were dispersed in ethanol and ultrasonicated for 5–10 min. A small drop of the sample was then taken on a carbon coated copper grid and dried before viewing.

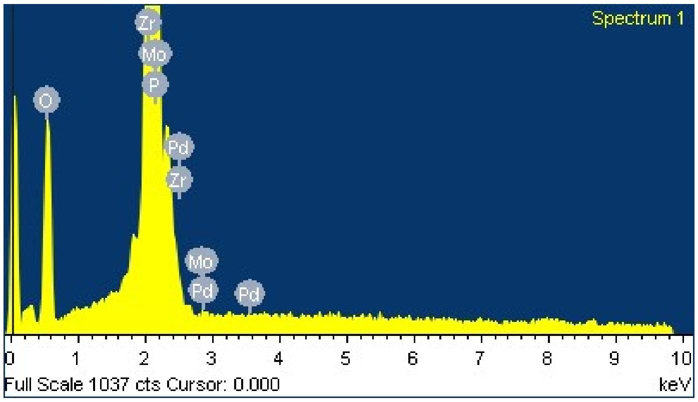

Figure S1. EDX of Pd-PMA/ZrO_2

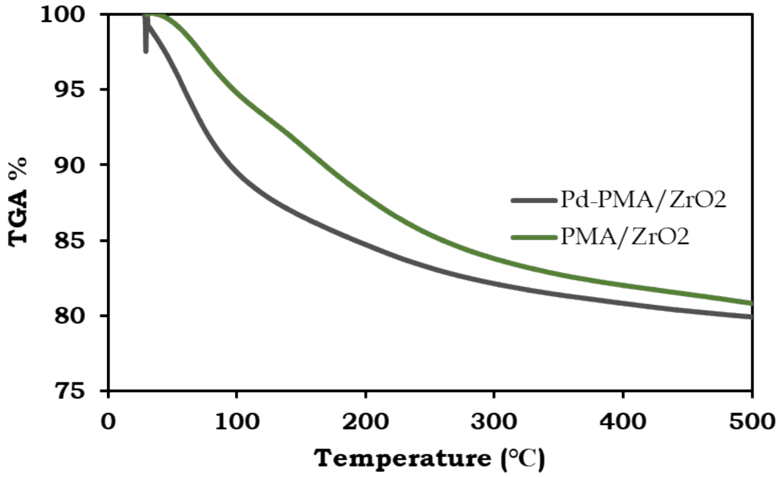

Figure S2. TGA of Pd-PMA/ZrO_2

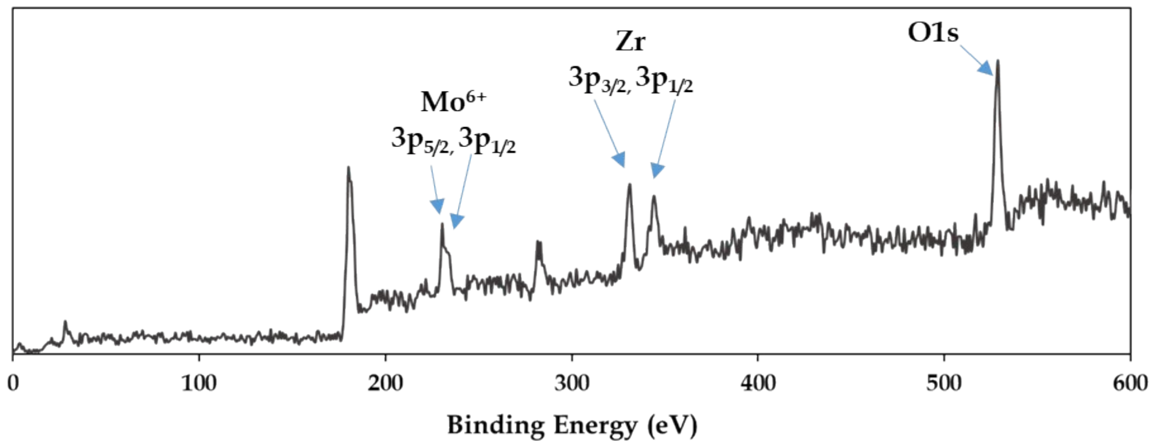

Figure S3. XPS of Pd-PMA/ZrO_2

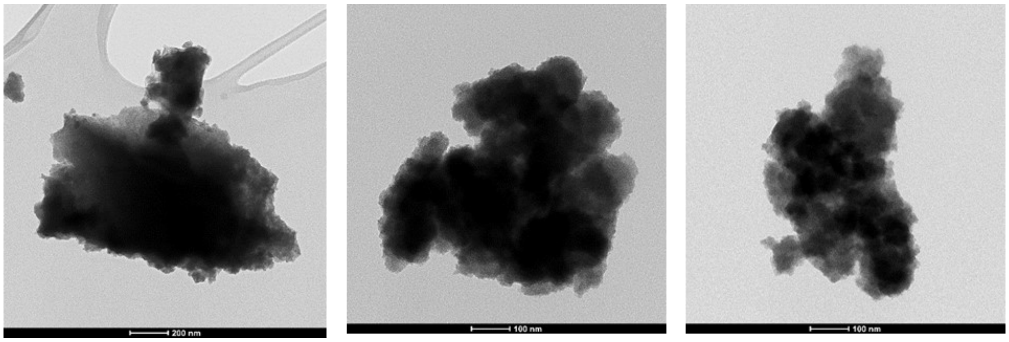

Figure S4. TEM images of PMA/ZrO_2

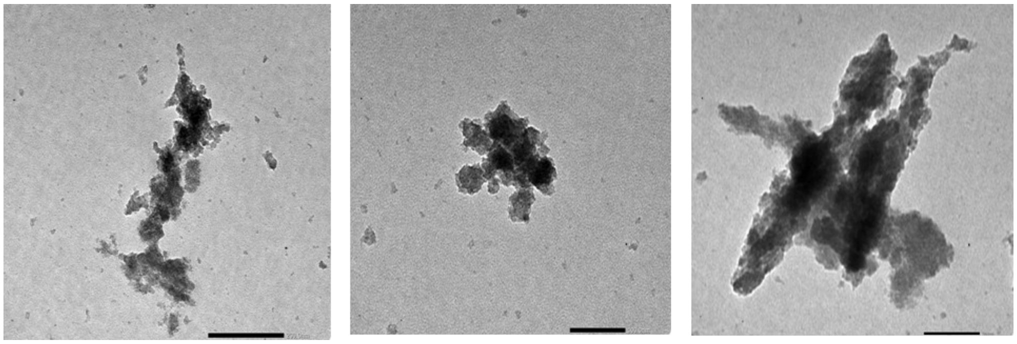

Figure S5. TEM images of Pd/ZrO_2

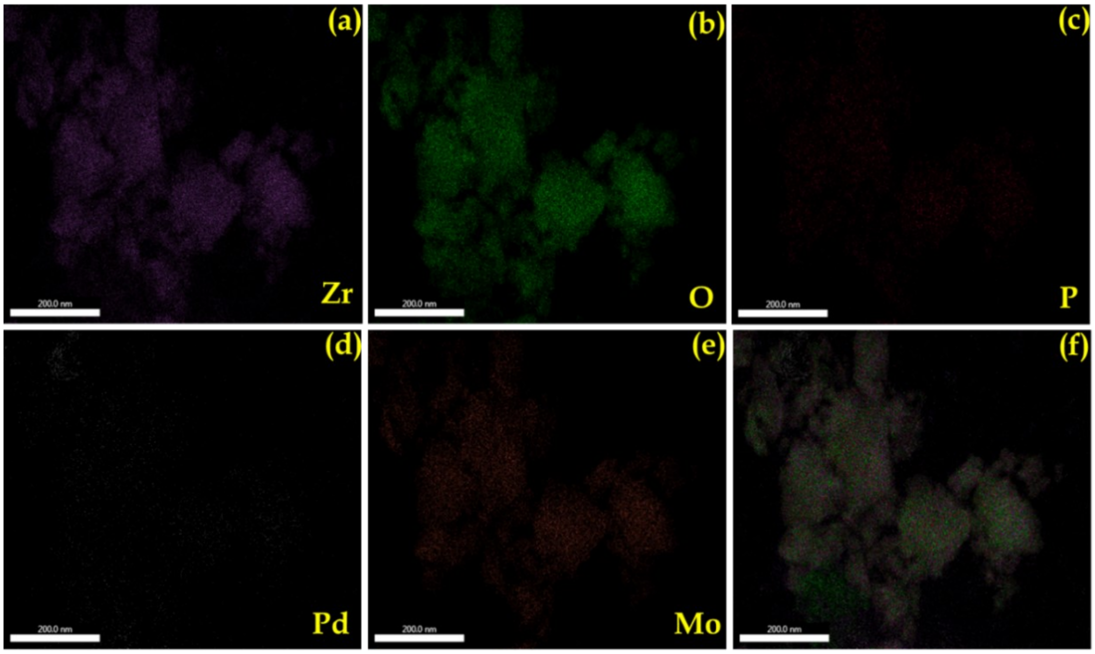

Figure S6. (a-e) elemental and (f) overlapping images of Pd-PMA/ZrO_2

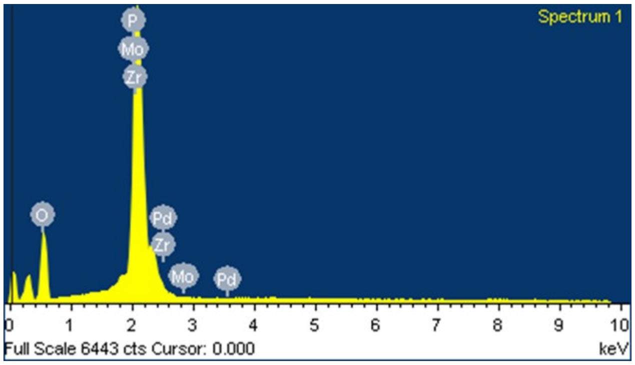

Figure S7. Elemental mapping of R-Pd-PMA/ZrO_2

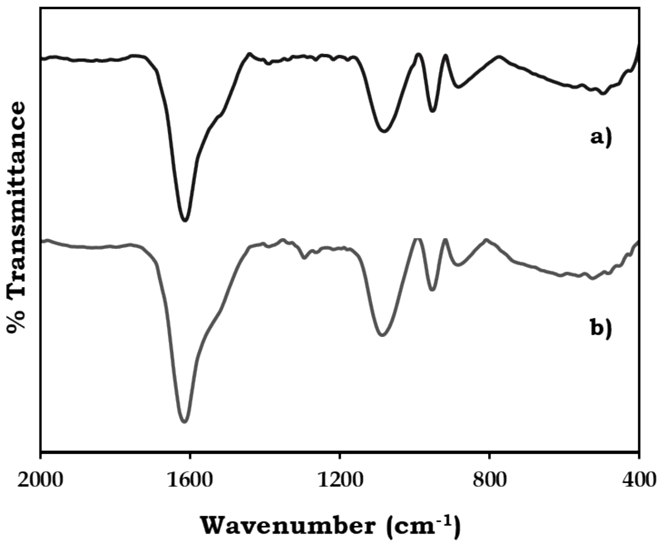

Figure S8. FT-IR of a) R-Pd-PMA/ZrO_2 b) F-Pd-PMA/ZrO_2

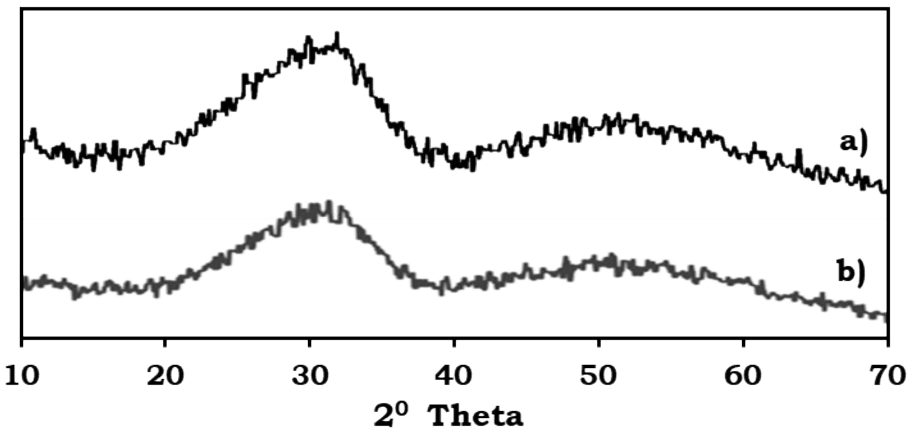

Figure S9. Powder XRD of a) R-Pd-PMA/ZrO_2 b) F-Pd-PMA/ZrO_2

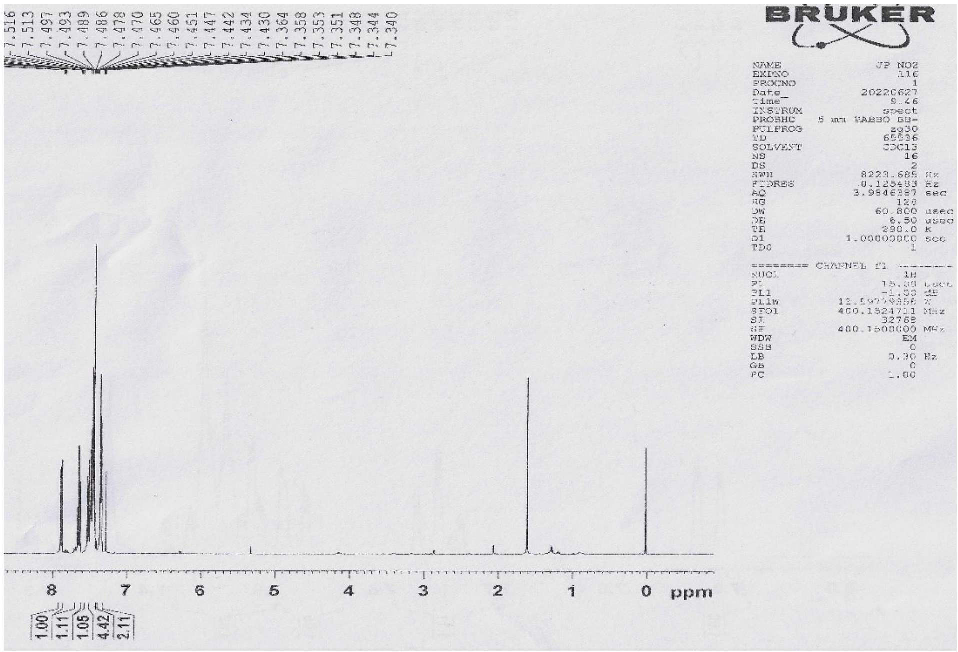

Figure S10. ^{1}H NMR of 2-nitrobiphenyl

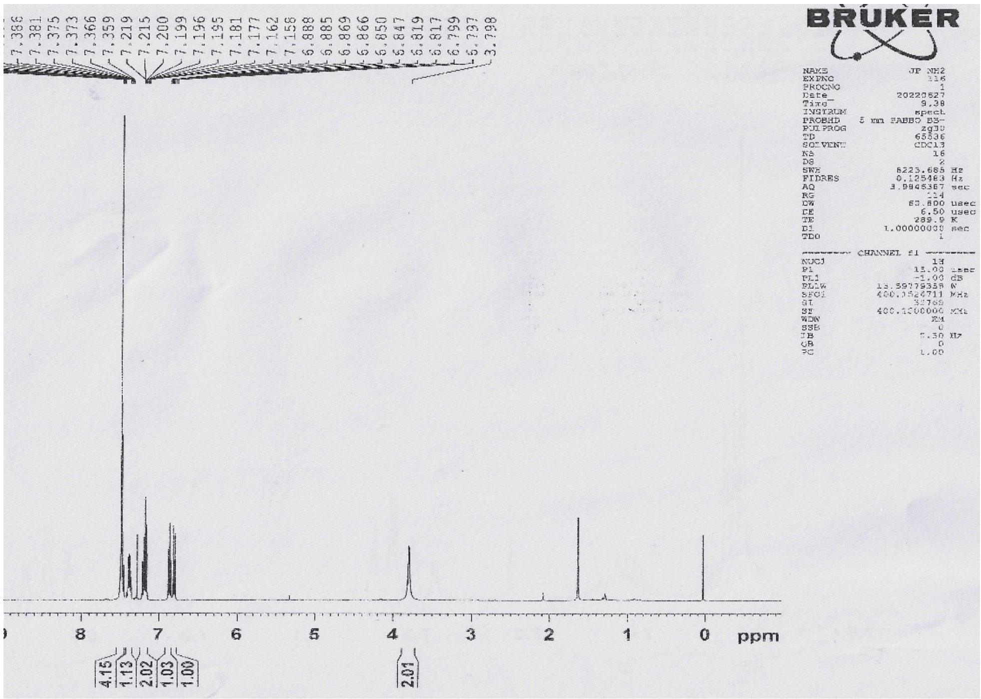

Figure S11. ^{1}H NMR of 2-aminobiphenyl

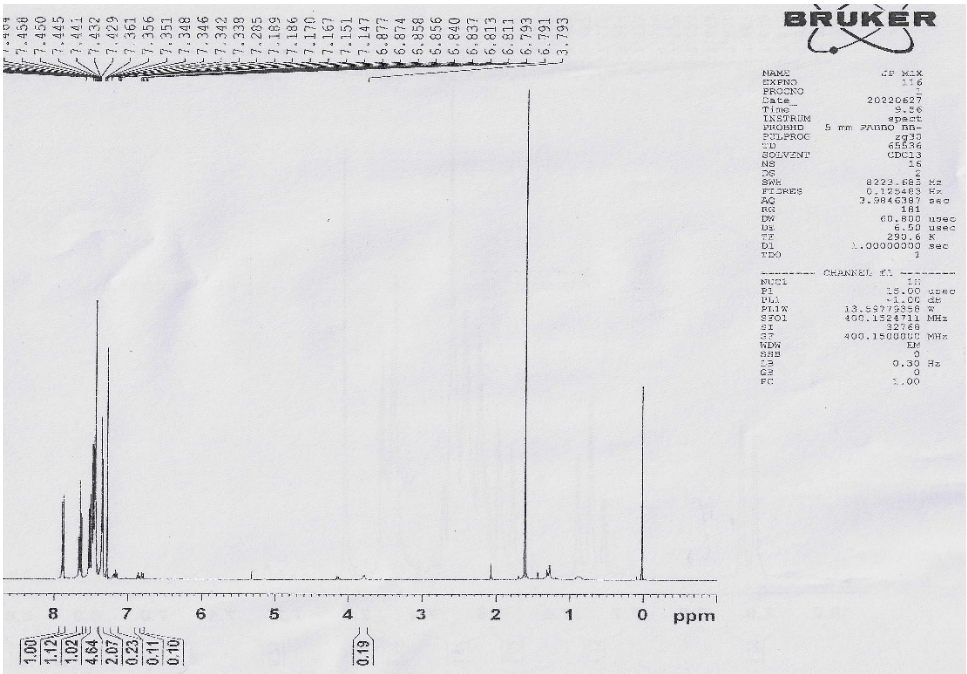

Figure S12. ^{1}H NMR of 10 h reaction mass
